# Is preference for mHealth intervention delivery platform associated with delivery platform familiarity?

**DOI:** 10.1186/s12889-016-3316-2

**Published:** 2016-07-22

**Authors:** Daniel Granger, Corneel Vandelanotte, Mitch J. Duncan, Stephanie Alley, Stephanie Schoeppe, Camille Short, Amanda Rebar

**Affiliations:** Physical Activity Research Group, School of Human, Health and Social Sciences, Building 18, Central Queensland University, Rockhampton, QLD 4702 Australia; Priority Research Centre for Physical Activity and Nutrition, University of Newcastle, Advanced Technology Centre, University Drive, Callaghan, NSW 2308 Australia; South Australian Health and Medical Research Institute, Faculty of Health Sciences, The University of Adelaide, North Terrace, Adelaide, 5000 South Australia Australia

**Keywords:** Smartphone, Tablet, Web-based, mHealth, Online, Internet, Delivery mode

## Abstract

**Background:**

The aim of this paper was to ascertain whether greater familiarity with a smartphone or tablet was associated with participants’ preferred mobile delivery modality for eHealth interventions.

**Methods:**

Data from 1865 people who participated in the Australian Health and Social Science panel study were included into two multinomial logistic regression analyses in which preference for smartphone and tablet delivery for general or personalised eHealth interventions were regressed onto device familiarity and the covariates of sex, age and education.

**Results:**

People were more likely to prefer both general and personalised eHealth interventions presented on tablets if they reported high or moderate tablet familiarity (compared to low familiarity) and people were more likely to prefer both general and personalised eHealth interventions presented on smartphones if they reported high or moderate smartphone familiarity, were younger, and had university education (compared to completing high school or less).

**Conclusion:**

People prefer receiving eHealth interventions on the mobile devices they are most familiar with. These findings have important implications that should be considered when developing eHealth interventions, and demonstrates that eHealth interventions should be delivered using multiple platforms simultaneously to optimally cater for as many people as possible.

## Background

Across the developed world, two-thirds of recorded deaths are attributed to preventable chronic diseases such as cardiovascular and respiratory diseases, obesity, diabetes, and depression [1]. The majority of Australians are currently living with at least one of these diseases [2] and approximately 50 % of the government’s health budget is expended treating these preventable health conditions [2–4]. The risk of developing chronic diseases is greatly enhanced by engaging in unhealthy behaviours [5], including smoking, poor diet, excessive alcohol consumption and insufficient physical activity [1, 6]. Positive health behaviour changes significantly reduce the risk of developing chronic diseases [5, 7]. Public health campaigns promoting healthy lifestyles are one way for alleviating the strain that these chronic diseases place upon people and the health care system [8].

The Internet has become an increasingly popular mode of communication, both in terms of awareness raising as well as in terms of behavioural modification [9], and has changed how people interact with information [10]. In the field of public health the use of web-based tools to exchange health information is often referred to as eHealth [11, 12]. Since 75 % of people with chronic diseases access treatment and management information from web-based services, eHealth applications are becoming increasingly attractive for exchanging health information and conducting behavioural interventions [13].

Although a large amount of research conducted on eHealth behaviour change interventions has shown to be effective to some extent [14–16], there are several studies that have shown ambiguous or no substantial effect of health behaviour programs [17, 18]. The heterogeneity in effectiveness of eHealth interventions may be partially attributable to the experiences people have using the wide range of devices that can deliver behaviour change eHealth interventions (e.g., personal computer, tablet, smartphone). The mixed results may indicate that eHealth behaviour change interventions need to be delivered in a way that aligns with previous user experiences in order to be effective. Market research has shown that there is a wide variety of preferences for the use of technological devices [19], but little is known regarding whether people have different preferences for the mode of technology used to deliver eHealth interventions [20]. Most behavioural eHealth interventions deliver intervention content only through one mode or type of device, and this may be limiting in terms of both reach and effectiveness.

It may be that people prefer to use the mobile devices they are most familiar with for eHealth interventions (i.e., *mHealth*). The cognitive demands required to navigate unfamiliar eHealth technology may be high and interfere with a person’s ability to change their health behaviour [21]. The wide variety of modalities offer many opportunities to reach people, though a greater understanding is needed of what mobile modalities work best for whom. Previous research suggests that considering people’s preferences when developing interventions is important as it ensures higher participant retention rates and enables participants a sense of autonomy [22, 23].

Currently there is a shifting trend from PC-based to mobile-based eHealth modes of delivery (e.g., smartphones, tablets) [24]. The ubiquity of internet-capable mobile devices has made tablets and smartphones an integral part of daily life for many people, and researchers are increasingly using these tools to promote healthy behaviours [25–27]. However, comparative studies of preferences between these devices are lacking. Given the increasing popularity of smartphones and tablets, information on preferences for mHealth delivery on tablets and smartphones is important information for health intervention developers.

The aim of this paper was to ascertain whether greater familiarity with a smartphone or tablet was associated with participants’ preferred delivery modality for mHealth interventions. Current eHealth interventions vary greatly in how information is presented to people; therefore the study investigated two outcomes: preferences for delivery of general health information and preferences for delivery of health information personally tailored to them. It was hypothesised that people would prefer the delivery of eHealth information on mobile devices they are most familiar with. Technology use tends to vary as a function of demographic characteristics [28]; therefore age, sex, and education were accounted for in the hypothesis testing.

## Method

### Study population

Study participants were members of the Australian Health and Social Science (AHSS) panel study conducted by the Population Research Laboratory at Central Queensland University. AHSS panel study members were a randomly selected sample of Australian adults recruited through annual population surveys conducted by the Population Research Laboratory. Panel members provided concent to complete regular online surveys on various health-related topics. Between October and November 2013, 3901 panel members across all States and Territories in Australia were invited to participate in an online survey for the current study. Of these, 2034 respondents (52.1 %) completed the survey. Participants provided informed consent and the Human Ethics Committee at Central Queensland University approved the study (H13/09-163).

### Measures

#### Preference for delivery of eHealth interventions

Using two items, participants reported their preferred device for the delivery of general (‘*What is your preferred platform for delivery of general health information?’*) and personalised *(‘What is your preferred platform for delivery of personalised health information?’*) health information from the options ‘through a desktop computer with internet access,’ ‘through a laptop computer with internet access,’ ‘through a computer-tablet via the internet, ‘through a telephone call,’ ‘through video conferencing,’ ‘through a mobile phone,’ ‘through a smartphone via the internet,’ ‘through a smartphone via an application,’ through e-mail,’ or ‘I do not want to use technology to receive disease management or health information’. Since this study aim focused on smartphones and tablet preferences, responses were collapsed into the categories of ‘*smartphone,’ ‘tablet,’* and *‘other.’*

#### Device familiarity

Device familiarity for smartphones and tablets was assessed using the questions, ‘*How often do you use smartphone applications?*’ and ‘*How often do you use tablet applications?*’ Based on response frequencies, responses were collapsed into the categories high (*‘at least once every hour’* or *‘several times each day’, ‘once a day’*), moderate (‘*several times each week*’ and ‘*once a week’*) and low (‘*once a month,’* ‘*less than once a month,’* ‘*never,*’ or *‘do not own the device’*).

#### Demographic covariates

Participants also reported their sex (*female* or *male*), age in years, and education (11 response options). Based on response frequencies, education was categorized into the nominal categories of: high school completion or less, university education (including bachelors, masters or PhD), and technical studies (e.g., trade certificate).

### Data analyses

Two multinomial logistic regression analyses were performed in which preference for smartphone or tablet delivery of general or personal eHealth interventions were regressed onto tablet familiarity, smartphone familiarity, and the covariates of sex, age, and education. Significance was set at *p* < 0.05. The data was analysed using R version 31 [29].

## Results

Of 2034 participants included in the study, 169 were excluded from analyses because they did not respond to any of the questions used in this study leaving a sample size of 1865. Amongst the sample, there was minimal missing data (age: *missingness n* = 9, 0.5 %; education: *missingness n* = 10, 0.5 %). Study sample characteristics are shown in Table 1. Half of participants were female (52.3 %) and most were older than 50 years (*M* = 55.96, *SD* = 13.68). Most participants completed some university schooling (i.e., obtained a bachelors, masters, or PhD). The majority of participants had low smartphone (low: *n* = 979, 52.5 %; moderate: *n* = 219, 11.7 %; high: *n* = 667, 35.8 %) and tablet familiarity (low: *n* = 1155, 61.9 %; moderate: *n* = 244, 13.1 %; high: *n* = 466, 25.0 %). When asked which device they would prefer to receive general health information on, most reported preferring non-mobile devices (desktop computer: 24.2 %, *n* = 451; laptop computer: 15.8 %, *n* = 295). However, 12.9 % (*n* = 241) of participants said smartphones and 10.2 % (*n* = 191) of participants said tablets. Response frequencies were similar in regards to which devices participants would prefer to receive personalised health information on: 19.9 % (*n* = 371) said desktop computers, 13.2 % (*n* = 246) said laptops, 10.1 % (*n* = 188) said smartphones and 10.2 % (*n* = 191) said tablets. A full break-down of the participants’ preferences are reported in Table 2.

**Table 1:** Study sample characteristics

Sex		
Female	*n* = 975	52.3 %
Male	*n* = 890	47.7 %
Age	*M* = 56.0	*SD* = 13.7
Education		
High School Completion or Less	*n* = 433	23.3 %
University Education (bachelors, masters, or PhD)	*n* = 1147	61.8 %
Technical Studies (e.g., trade certificate)	*n* = 275	14.8 %

*Notes*: Missing data for age (*n* = 9, 0.5 %) & education (*n* = 10, 0.5 %)

**Table 2:** Participants’ reported preferences for receiving general and personalised health information

	Generalised		Personalised	
	*n*	*%*	*n*	*%*
Tablet	241	12.9 %	188	10.1 %
Smartphone	191	10.2 %	191	10.2 %
Desktop computer	451	24.2 %	371	19.9 %
Laptop computer	295	15.8 %	246	13.2 %
Telephone call	43	2.3 %	81	4.3 %
Video conferencing	11	0.6 %	19	1.0 %
Mobile phone	52	2.8 %	87	4.7 %
E-mail	214	11.5 %	298	16.0 %
I do not want to use technology	367	19.7 %	384	20.6 %

### Preferred device for general health information

The results of the multinomial logistic regression for tablet and smartphone preference for receiving general health information are presented in Table 3.

**Table 3:** Summary of multinomial logistic regression model for device familiarity predicting general health intervention device preferences, controlling for sex, age, and education

	Tablet preference (compared to other)			Smartphone preference (compared to other)		
	*B*	*SE B*	*e* ^*B*^	*B*	*SE B*	*e* ^*B*^
Intercept	−3.53*	0.48	--	−3.52*	0.67	--
Tablet familiarity						
Reference: Low familiarity						
High familiarity	3.30*	0.24	27.09	0.34	0.21	1.41
Moderate familiarity	2.03*	0.28	7.60	0.13	0.25	1.14
Smartphone familiarity						
Reference: Low familiarity						
High familiarity	0.46*	0.19	1.59	3.93*	0.52	51.10
Moderate familiarity	−0.00	0.28	1.00	2.67*	0.57	14.39
Sex						
Reference: Female						
Male	−0.30	0.17	0.74	0.12	0.18	1.13
Education						
Reference: High school degree or less						
University education	0.52*	0.22	1.69	1.22*	0.29	3.38
Technical studies	0.13	0.31	1.14	0.60	0.38	1.82
Age	−0.01	0.01	1.00	−0.05*	0.01	0.95

*Note:* Residual deviance = 1791.65, AIC = 1827.65. *p < .05, *e* ^*B*^ = exponentiated *B*

#### Preference for tablets

People with high and moderate tablet familiarity were more likely to prefer receiving general health information on tablets compared to other devices. Additionally, people who had high smartphone familiarity were more likely to prefer the tablet than people with low familiarity. People with university schooling were more likely to report a preference for tablets compared to people with a high school degree or less. Preference for tablets did not significantly differ as a function of sex or age.

#### Preference for smartphones

People with high and moderate smartphone familiarity were more likely to prefer receiving general health information on smartphones compared to other devices. People with university schooling were more likely to report a preference for smartphones compared to people with a high school degree or less. Additionally, younger people were more likely to report a smartphone preference than older people. Preference for smartphones did not significantly differ as a function of tablet familiarity or sex.

### Preferred device for personal health information

The results of the multinomial logistic regression for tablet and smartphone compared to other devices for delivery of personalised health information are presented in Table 4.

**Table 4:** Summary of multinomial logistic regression model for device familiarity predicting personalised health intervention device preferences, controlling for sex, age, and education

	Tablet preference (compared to other)			Smartphone preference (compared to other)		
	*B*	*SE B*	*e* ^*B*^	*B*	*SE B*	*e* ^*B*^
Intercept	−4.12*	0.54	--	−2.67*	0.55	--
Tablet familiarity						
Reference: Low familiarity						
High familiarity	3.41*	0.27	30.14	0.32	0.20	1.38
Moderate familiarity	2.05*	0.33	7.74	0.35	0.24	1.43
Smartphone familiarity						
Reference: Low familiarity						
High familiarity	0.20	0.21	1.23	3.13*	0.38	22.95
Moderate familiarity	0.00	0.30	1.00	1.90*	0.45	6.71
Sex						
Reference: Female						
Male	−0.48*	0.18	0.62	−0.04	0.12	0.96
Education						
Reference: High school degree or less						
University education	0.44	0.24	1.56	0.86*	0.27	2.36
Technical studies	0.33	0.33	1.40	0.45	0.36	1.57
Age	−0.00	0.01	1.00	−0.05*	0.01	0.95

*Note:* Residual deviance = 1718.54, AIC = 1754.54. *p < .05, *e* ^*B*^ = exponentiated *B*

#### Preference for tablets

People with high and moderate tablet familiarity were more likely to prefer receiving personalised health information on tablets compared to other devices. Additionally, men were less likely to report a preference for tablets compared to women. Preference for tablets did not significantly differ as a function of smartphone familiarity, education, or age.

#### Preference for smartphones

People with high and moderate smartphone familiarity were more likely to prefer receiving personalised health information on smartphones compared to other devices. People with university schooling were more likely to report a preference for smartphones compared to people with a high school degree or less. Additionally, younger people were more likely to report a smartphone preference than older people. Preference for smartphones did not significantly differ as a function of tablet familiarity or sex.

## Discussion

The aim of this study was to ascertain whether people would prefer delivery of mHealth interventions on a device that was familiar to them. The results supported our hypothesis – greater familiarity with either smartphones or tablets was associated with a higher preference for that device for mHealth delivery. Given that eHealth interventions are most effective when they take people’s preferences into account [20, 22, 23]; these findings suggest mHealth interventions may be more effective when intervention delivery methods align the familiarity people have in using these intervention delivery methods.

In accordance with the hypothesis, people who were highly familiar with a tablet were more likely to prefer health information delivered via a tablet (for both general and personalised information) than people who were unfamiliar with tablets. Similarly, high frequency users of smartphone applications were more likely to prefer health information delivered via their smartphone (for both general and personalised information). Thoma and Williams [30] showed that people self-identify with devices that are familiar to them, which may mean that the use of these devices requires less cognitive resources [21]. Using a familiar device is not as cognitively demanding as having to navigate a novel device; therefore having intervention participants use devices they are familiar with, and which they prefer, may thus allow for a greater cognitive capacity for behaviour change.

Of note, more participants reported a preference for non-mobile devices (i.e., desktop or laptop computers) for delivery of eHealth interventions, though one should keep in mind that the average age of this sample was 56 years. This is probably due to lack of familiarity of using mobile devices for eHealth in this sample. This is in contrast to the rapid transformation of the field in incorporating mobile devices for eHealth [24]. As such, researchers should consider providing eHealth interventions with accessibility across mobile and non-mobile devices. Alternatively, more consideration is needed to familiarize people with mobile devices within mobile eHealth interventions. This is an important behaviour change barrier that can easily be overcome during intervention implementation. For example, there could be an initial face-to-face session where participants are familiarised with the mHealth intervention on their own device. This practice was frequently applied when web-based interventions were still new [16]. Alternatively, mHealth interventions could incorporate peer-to-peer communication features (e.g., social networking), so that participants can help one another in gaining optimal knowledge as how to best use the mHealth intervention [31].

Younger people and people with university education were more likely to prefer general health information delivered to a smartphone. These findings are consistent with the results from a recent study showing that highly educated millennials are more familiar using smartphones for interacting within their professional and social worlds than their peers [32]. This suggests that special consideration is needed for older or less educated intervention participants to ensure they receive sufficient training to improve their familiarity with these devices, or alternatively that computers are used, rather than mobile devices, for interventions in these groups.

### Limitations & future directions

This study utilised a self-report method of data collection which has limitations [33]. Social desirability biases may result in inflated self-reported technology use. Furthermore, the survey was cross-sectional, and, as such, it cannot inform causal inferences [33]. Future research, that can address the issue of causality, should use longitudinal designs, as well as objectively monitor technology use to address this. The findings reflect those of a population at a certain point in history; eHealth technology is changing and developing at a high pace, and research will need to continue to test for cohort effects of these findings as technology advances and the general population becomes more familiar with the devices. For example, eHealth interventions have begun to incorporate virtual reality simulations [34–36]. It will be important to determine whether people will feel uncomfortable utilizing this technology or if the novelty can enhance the engagement experience. This study focused on health information delivery; however future research on the delivery device preferences for cognitive behavioural interventions could yield valuable information with regards to intervention efficiency and efficacy. For example, cognitive behaviour therapy and cognitive bias modification therapy have already been shown to be successfully delivered through online formats [37]. Developers in these fields would thus benefit from considering the effects of device preference and familiarity when designing new interventions.

## Conclusion

This study demonstrates that device familiarity significantly impacts device preference for eHealth interventions. People in this study tended to report preferring non-mobile devices for eHealth interventions overall, suggesting that these may still need to be options for the delivery of eHealth interventions. Amongst the mobile devices, a preference for smartphones was found to be more likely amongst younger and more educated populations. These findings are important for improving the methods by which state health agencies, non-government organizations, health promoters, health insurance companies, and intervention developers deliver health interventions to the public. The field of eHealth has already had a significant impact on increasing health literacy and improving health behaviours. The current literature has confirmed the utility of smartphones and tablets in delivering health behaviour change interventions, and the findings of this study suggest these effects might be enhanced by providing eHealth interventions using the devices people are familiar with.
